# Results of Total Knee Arthroplasty with Robotic Assistance *

**DOI:** 10.1055/s-0045-1814408

**Published:** 2025-12-30

**Authors:** Joao Paulo Fernandes Guerreiro, Julia Canassa Pinto, Livia Schauff, Tiago Delfino Pedrollo, Paulo Roberto Bignardi, Marcus Vinicius Danieli

**Affiliations:** 1 Pontifícia Universidade Católica do Paraná, Londrina, PR, Brazil; 2 Hospital de Ortopedia Uniorte, Londrina, PR, Brazil

**Keywords:** arthroplasty, blood loss, surgical, knee, robotic surgical procedures, artroplastia, joelho, perda sanguínea cirúrgica, procedimentos cirúrgicos robóticos

## Abstract

**Objective:**

To investigate the outcomes of total knee arthroplasty (TKA) when assisted by a robotic arm compared with the conventional approach.

**Methods:**

We conducted a retrospective cohort study including 96 patients who underwent TKA, assigned to either the robotic-assisted (RA) group or a conventional technique (CT) groups. All surgeries were performed without the use of a tourniquet and included administration of intravenous tranexamic acid. Patients were matched based on sex, age, and preoperative hemoglobin (Hb) and hematocrit (Ht) values. Key outcomes evaluated were perioperative blood loss (assessed through changes in Hb and Ht), operative duration, length of hospital stay, and postoperative complications up to 6 months.

**Results:**

There were 34 patients from each group successfully matched for analysis. There were no significant differences between the groups regarding Hb or Ht reduction (RA: Hb −2.27 ± 1.21 g/dL, Ht −6.56 ± 3.43%; vs. CT: Hb −2.00 ± 1.07 g/dL, Ht −5.85 ± 3.26%; *p* > 0.05). The mean surgical time was also similar (RA: 108.9 ± 20.8 vs. CT: 111.8 ± 26.2 min; *p* = 0.905). Notably, patients in the RA group experienced a shorter hospitalization period (median: 2 vs. 2.5 days; OR = 0.12; 95% CI = 0.03–0.57; *p* = 0.008). Incidence of postoperative complications within 6 months did not differ significantly between groups.

**Conclusion:**

Robotic-assisted TKA was not associated with measurable improvements in blood loss, operative time, or postoperative complications. However, it contributed to a reduction in hospital stay compared with the conventional technique.

## Resumo

**Objetivo:**

Investigar os resultados da artroplastia total do joelho (ATJ) com assistência de braço robótico em comparação com a abordagem convencional.

**Métodos:**

Realizamos um estudo de coorte retrospectivo, incluindo 96 pacientes submetidos a ATJ, que foram alocados aos grupos assistência robótica (AR) ou técnica convencional (TC). Todas as cirurgias foram realizadas sem o uso de torniquete e incluíram a administração de ácido tranexâmico intravenoso. Os pacientes foram pareados com base no sexo, na idade e nos valores pré-operatórios de hemoglobina (Hb) e hematócrito (Ht). Os principais desfechos avaliados foram a perda sanguínea perioperatória (avaliada por meio de alterações em Hb e Ht), a duração operatória, o tempo de internação hospitalar e as complicações pós-operatórias até em 6 meses.

**Resultados:**

Foram 34 pacientes para cada grupo pareados com sucesso para análise. Não houve diferenças significativas entre os grupos quanto à redução de Hb ou Ht (AR: Hb −2,27 ± 1,21 g/dL, Ht −6,56 ± 3,43%; vs. TC: Hb −2,00 ± 1,07 g/dL, Ht −5,85 ± 3,26%; *p* > 0,05). O tempo cirúrgico médio também foi semelhante (AR: 108,9 ± 20,8 vs. TC: 111,8 ± 26,2 min; *p* = 0,905). Notavelmente, os pacientes do grupo AR tiveram um período de hospitalização mais curto (mediana: 2 vs. 2,5 dias; OR = 0,12; IC 95% = 0,03–0,57; *p* = 0,008). As complicações pós-operatórias em 6 meses não diferiram significativamente entre os grupos.

**Conclusão:**

A ATJ com assistência robótica não foi associada a melhorias mensuráveis na perda sanguínea, tempo operatório ou complicações pós-operatórias. Entretanto, contribuiu para uma redução na permanência hospitalar em comparação com a técnica convencional.

## Introduction

Total knee arthroplasty (TKA) is a common surgical intervention aimed at relieving pain and restoring function in patients with severe osteoarthritis or other debilitating joint conditions. 1 This procedure involves replacing the knee joint with a prosthesis by removing damaged portions of the femur, tibia, and cartilage. 2 However, TKA is associated with significant concerns regarding bleeding, which may lead to postoperative complications, increase the risk of transfusions, and prolong patient recovery. 2

In recent decades, the introduction of robotic arm assistance has revolutionized the precision and effectiveness of the procedure, raising important questions about its impact on bleeding levels. 3 Conventional TKA relies on preoperative radiographs, intraoperative anatomical landmarks, and manual alignment guides, whereas robotic-assisted TKA provides a virtual three-dimensional (3D) reconstruction that accounts for each patient's specific anatomy. 4 5 This enables more accurate planning of bone resections and prosthesis positioning, thereby improving tissue preservation and reducing postoperative inflammation and pain, 4 5 although no significant differences have been demonstrated in medium- and long-term functional outcomes when compared with the conventional technique. 6 7 8 9 10

Additionally, other perioperative strategies, such as the use of the antifibrinolytic agent tranexamic acid, have been employed to reduce blood loss and the need for transfusions. 11 Several meta-analyses have shown that tranexamic acid is safe and significantly decreases both total and postoperative blood loss, as well as the mean number of transfusions per patient, without increasing the rate of complications. 12 13 14

Tourniquets have been traditionally used in TKA, based on the belief they can reduce intraoperative bleeding and improve prosthesis cementation. 15 However, a meta-analysis published in 2021 indicated that tourniquet use may not offer significant advantages in this regard and is associated with risks, such as soft-tissue damage, greater postoperative pain, and longer hospital stay. Although it may reduce surgical time, these potential adverse effects highlight the need to reconsider its routine use. 15

To date, there is no consensus regarding bleeding rates in robotic-assisted TKA, particularly when performed in combination with intravenous tranexamic acid and without the use of a tourniquet. 16

The hypothesis of this study is that robotic-assisted TKA results in lower perioperative bleeding, fewer postoperative complications, and reduced surgical time when compared with the conventional surgical method.

## Materials and Methods

### Ethical Aspects

This study was conducted after approval by the Institutional Research Ethics Committee, CAAE number 79863824.2.0000.5696.

### Study Design

This was a retrospective observational study performed through the analysis of medical records of patients who underwent TKA performed by the same knee surgery team between July 2022 and 2023. Patients were divided into two distinct groups: one in which TKA was performed with robotic-assistance and the other with the conventional method.

Inclusion criteria comprised all patients who underwent TKA without the use of a tourniquet and with administration of tranexamic acid. Exclusion criteria were revision TKA, concomitant corrective osteotomy, previous coagulation disorders, or use of anticoagulant medication within 7 days prior to surgery.

## Data Collection

The following data were collected from patient medical records during hospitalization: sex, age, weight, height, previous coagulation disorders, date of surgery, surgical time, robotic-assistance, as well as use of anticoagulant medications, tranexamic acid, and tourniquet. We also collected data on intraoperative complications, need for additional procedures, preoperative and 24-hour postoperative Hb and Ht levels, length of hospital stay, and postoperative complications up to the 6th month, the latter of which included transfusions, reoperation for hematoma drainage, arthrofibrosis, wound dehiscence, superficial and deep infection, deep vein thrombosis (DVT), pulmonary embolism (PE), myocardial infarction, and death.

All patients included had blood samples collected through peripheral venipuncture in the preoperative period and at 24 hours after surgery. Surgical time for each procedure was recorded by the nursing and anesthesia teams. All patients underwent spinal anesthesia and received 1 g of intravenous tranexamic acid at induction. The medial parapatellar approach was preferred, without tourniquet use.

The implants were cemented, without patellar replacement, and with the same instrumentation. In the robotic-assisted group, the ROSA Knee System (Zimmer Biomet) was used. No suction drain was applied in any case.

Thromboprophylaxis consisted of mechanical methods using an intermittent pneumatic compression device on the calves during the first 24 hours postoperatively, along with elastic compression stockings for the first 30 days. Pharmacological prophylaxis included rivaroxaban 10 mg orally, initiated 6 hours after the procedure and maintained at the same dose every 24 hours for 10 days in all patients.

The hospital discharge criteria included hemodynamic stability, adequate pain control, and the ability to ambulate independently with a walker.

### Statistical Analysis

Both Hb and Ht were considered dependent variables, while age, sex, surgical time, length of hospital stay, and complications (including death) were considered independent ones. Categorical variables were expressed as frequency and percentage, while quantitative ones were assessed for normality using the Shapiro–Wilk test. The statistical significance of qualitative variables was evaluated using Fisher's exact test, and quantitative variables were analyzed with the Student's *t* or Mann–Whitney's tests, according to data distribution.

Additionally, a multivariate regression analysis adjusted for age and sex was performed using the Wald test to determine the impact of robotic-assistance on reductions in Hb and Ht, surgical time, hospital stay, and complication rates. All analyses were performed using the Stata/SE v.16.1 (StataCorp LLC), with the significance level set at *p* < 0.05.

## Results

**Fig. 1:**
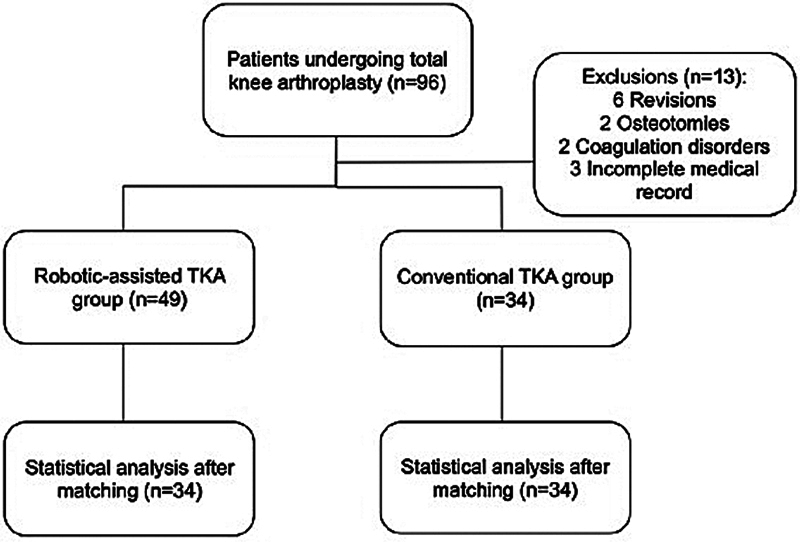
Study's flowchart.

During the study period, a total of 96 patients underwent TKA without tourniquet use and with tranexamic acid administration. Of these, 85 patients were included in the study; furthermore, 6 were excluded due to revision surgery, 2 due to associated osteotomy, and 2 due to coagulation disorders). After statistical matching of the groups, the robotic-assisted and conventional surgery groups comprised 34 patients each. The study flowchart is illustrated in Fig. 1 .

Table 1 presents the baseline characteristics of the patients. The mean age, sex distribution, and baseline Hb and Ht levels were similar between the groups, with no statistically significant differences ( *p* > 0.05).

**Table 1:** Baseline characteristics of the patients

	Robotic-assisted ( *n* = 34)	Conventional ( *n* = 34)	*p* -value
Age (years)	67.8 ± 9.7	68.2 ± 7.4	0.845 ^a^
Males, n (%)	26 (53.1)	13 (38.2)	0.263 ^b^
Hb (g/dl)	13.56 ± 1.05	13.05 ± 1.31	0.079 ^a^
Ht (%)	40.32 ± 3.15	39.49 ± 4.19	0.358 ^a^
Time of surgery (min)	108.9 ± 20.8	111.79 ± 26.17	0.621 ^a^
Hospital stay (days)	2 (1–3)	2 (2–3)	0.008 ^c^
Transfusion (n)	0	0	
Superficial infection (n)	4 (11.8)	1 (2.9)	0.356 ^b^
Deep infection (n)	2 (5.6)	1 (2.9)	> 0.999 ^b^
Arthrofibrosis (n)	1 (2.9)	1 (2.9)	> 0.999 ^b^
Thrombosis (n)	0 (0)	1 (2.9)	> 0.999 ^b^
Embolism (n)		0	
Heart attack (n)	1 (2.9)	0 (0)	> 0.999 ^b^
Death (n)	0	0	

Abbreviations: Hb, hemoglobin; Ht, hematocrit. a Test t, b Chi-square, c Mann-Whitney test.

Surgical time and length of hospital stay were also evaluated. There was no significant difference in surgical time between the groups ( *p* = 0.621). However, there was a significant difference in hospital stay, with shorter lengths in the robotic-assisted group ( *p* = 0.008), as shown in Table 1 .

Regarding surgical complications, the results showed no statistically significant differences between the groups for any of the variables. No cases of PE, transfusion requirements, or deaths were identified ( Table 1 ).

As shown in Table 2 , the differences in mean pre- and postoperative (24h) Hb and Ht levels were not statistically significant between the robotic and conventional groups.

**Table 2:** Difference in means for Hb and Ht of patients undergoing TKA with and without the use of a robot in relation to the preoperative period

Time	Robotic-assisted Averages (SD)	Conventional Averages (SD)	*p* -value
Hb (mg/dl)			
24h	−2.27 (1.21)	−2.00 (1.07)	0.329
Ht (%)			
24h	−6.56 (3.43)	−5.85 (3.26)	0.383

**Abbreviation:** Hb, hemoglobin; Ht, hematocrit; SD, standard deviation; TKA, total knee arthroplasty.

In Table 3 , the multivariate regression analysis adjusted for age and sex revealed that robotic-assistance had a significant impact only on reducing hospital stay (OR = 0.12; 95% CI = 0.03–0.57; *p* = 0.008), with no significant influence on other variables (Hb, Ht, surgical time, and complications).

**Table 3:** Effect of robot use on reducing Hb and Ht, hospitalization days, TKA time, and presence of complications *

	OR	95% CI	*p* -value
Hb Reduction	1.83	0.67–4.99	0.236
Ht Reduction	0.91	0.65–1.23	0.594
Surgery time	1.01	0.98–1.03	0.905
Hospital stay	0.12	0.03–0.57	0.008
Superficial infection (n)	0.23	0.01–4.03	0.316
Deep infection (n)	2.19	0.08–60.1	0.642
Arthrofibrosis	0.78	0.04–15.0	0.868

**Abbreviation:** CI, confidence interval; Hb, hemoglobin; Ht, hematocrit; OR, odds ratio; TKA, total knee arthroplasty.

**Note:** *Multivariate regression analysis. Wald test. Model adjusted for age and sex.

## Discussion

This study did not demonstrate significant differences in the reduction of Hb and Ht levels after surgery between the robotic-assisted and conventional groups ( *p* > 0.05). This finding suggests that, in the absence of tourniquet use and with intravenous tranexamic acid administration, robotic-assisted TKA does not provide a clear advantage in terms of blood loss compared with the conventional technique.

These results are consistent with a retrospective study published in 2022, 16 which included 486 patients and employed both tourniquet and tranexamic acid. They also align with a systematic review and meta-analysis published in 2024, 17 which evaluated 12 studies involving 2,863 patients and analyzed transfusion requirements and blood loss in conventional versus robotic-assisted TKA. However, that review did not address the use of tranexamic acid or tourniquet in the included studies. 17

None of the patients in the present study required blood transfusion. In contrast, a retrospective cohort study by Khan et al., published in 2021, reported six cases of transfusion in the conventional group (12%) and one in the robotic-assisted group (2%), showing a statistically significant difference. 2 In their study, both tranexamic acid and a tourniquet were used in all patients. 2

Surgical time also did not differ significantly between groups in our study. The mean duration was 108.8 ± 20.8 minutes in the robotic-assisted group versus 111.79 ± 26.17 minutes in the conventional group ( *p* = 0.484). A previous randomized controlled trial reported that robotic-assisted TKA was associated with longer operative time compared with the conventional approach, 18 whereas a descriptive qualitative study suggested that robotic-assistance should reduce surgical time. 19 Robotic technology allows for preoperative simulations, preventing errors in prosthesis positioning and assisting surgeons' decision-making. 19 However, such procedures may sometimes take longer, particularly during the initial learning curve. 20 In our study, robotic-assisted surgeries were performed within the 1 ^st^ year after implementation of the system in our institution, yet no difference in surgical time was observed.

Postoperative complications classically described in TKA showed no significant differences between groups, 21 22 23 including transfusion, reoperation for hematoma drainage, arthrofibrosis, wound dehiscence, superficial and deep infection, DVT, PE, myocardial infarction, and death—. This is consistent with a retrospective cohort study published in 2022 analyzing a large TKA database, 24 which found no association of the increasing adoption of robotic-assisted TKA with higher rates of infectious or non-infectious complications.

Regarding infections, a retrospective study by Ofa et al., 25 published in 2020, compared robotic-assisted and conventional TKA and found no significant difference within 90 days postoperatively. Infection rates were 0.62% in the conventional group and 0.48% in the robotic-assisted group. 25 In our study, no statistical differences were observed, but the overall infection rate was higher than reported in the literature, reaching 4% at 6 months.

In terms of DVT, our study identified one case in the conventional group and none in the robotic-assisted group, without statistical significance. A retrospective study by Itou et al., published in 2023, 26 which used both a tourniquet and tranexamic acid, also found that robotic-assisted TKA was not associated with increased postoperative DVT risk. Another retrospective consecutive case series suggested that, despite longer operative times, robotic-assisted surgery may be safer, being associated with a lower incidence of DVT. 5 This may be attributed to less soft-tissue trauma and avoidance of femoral canal violation. 5

Furthermore, a randomized case-control study published in 2022 by Xu et al. 20 compared early clinical and radiographic outcomes of robotic-assisted versus conventional manual TKA with tourniquet use, reporting no significant differences in DVT incidence. Both studies emphasized the role of anticoagulant regimens, such as factor Xa inhibitors, in reducing postoperative DVT risk, consistent with our study protocol. 18 20 27

We found a significant reduction in hospital stay in the robotic-assisted group (95% CI: 0.03–0.57, *p* = 0.008), indicating a potential benefit of this technology in enhancing postoperative recovery. Previous studies comparing conventional and robotic-assisted TKA have also demonstrated shorter hospitalization times. 25 26 27 28 For instance, a retrospective cohort study published in 2024 reported a mean length of stay of 1.89 days in the robotic-assisted group versus 2.41 days in the conventional group. 28 Other retrospective studies published in 2018 and 2024 also found significant reductions in hospitalization duration for robotic-assisted TKA patients. 27 28

In our study, discharge criteria were based on hemodynamic stability, the ability to ambulate with a walker, and adequate pain control. It is noteworthy that the comparative studies reviewed here also reported tranexamic acid use during surgery but did not provide specific discharge criteria. Furthermore, in one study, 27 a pneumatic tourniquet was used but never inflated, while another 28 did not specify its use.

This study has several limitations. The most important being its retrospective design, which may have affected the results' accuracy. Moreover, being conducted in a single center limited the sample size. On the other hand, this approach minimized bias from inter-surgeon variability in technique. Additionally, no data on functional outcomes or pain scores were collected, precluding a more comprehensive comparison.

## Conclusion

In this study, the choice of surgical approach—robotic-assisted versus conventional TKA—did not result in significant differences in hemoglobin or hematocrit levels 24 hours after surgery. Similarly, operative time, the requirement for blood transfusions, and the incidence of postoperative complications were comparable between the two techniques.

Notably, patients undergoing robotic-assisted TKA had a significantly shorter hospital stay, indicating a potential advantage of this technology in promoting faster postoperative recovery and earlier discharge.

## References

[1] OrthoInfo Treatment: Total Knee Replacement American Academy of Orthopaedic Surgeons. Available from:https://orthoinfo.aaos.org/pt/treatment/artroplastia-total-de-joelho-total-knee-replacement/

[2] Khan H Dhillon K Mahapatra P Blood loss and transfusion risk in robotic-assisted knee arthroplasty: A retrospective analysis Int J Med Robot 2021 17 06 e2308 10.1002/rcs.2308 34288356

[3] Clark T C Schmidt F H Robot-assisted navigation versus computer-assisted navigation in primary total knee arthroplasty: efficiency and accuracy ISRN Orthop 2013 2013 794827 10.1155/2013/794827 24967115 PMC4045350

[4] Kayani B Konan S Ayuob A Onochie E Al-Jabri T Haddad F S Robotic technology in total knee arthroplasty: a systematic review EFORT Open Rev 2019 4 10 611 617 10.1302/2058-5241.4.190022 31754467 PMC6836078

[5] Siebert W Mai S Kober R Heeckt P F Technique and first clinical results of robot-assisted total knee replacement Knee 2002 9 03 173 180 10.1016/s0968-0160(02)00015-7 12126674

[6] Song E K Seon J K Park S J Jung W B Park H W Lee G W Simultaneous bilateral total knee arthroplasty with robotic and conventional techniques: a prospective, randomized study Knee Surg Sports Traumatol Arthrosc 2011 19 07 1069 1076 10.1007/s00167-011-1400-9 21311869

[7] Liow M HL Goh G SH Wong M K Chin P L Tay D KJ Yeo S J Robotic-assisted total knee arthroplasty may lead to improvement in quality-of-life measures: a 2-year follow-up of a prospective randomized trial Knee Surg Sports Traumatol Arthrosc 2017 25 09 2942 2951 10.1007/s00167-016-4076-3 27017214

[8] Yang H Y Seon J K Shin Y J Lim H A Song E K Robotic total knee arthroplasty with a cruciate-retaining implant: a 10-year follow-up study Clin Orthop Surg 2017 9 02 169 176 10.4055/cios.2017.9.2.169 28567218 PMC5435654

[9] Cho K J Seon J K Jang W Y Park C G Song E K Robotic versus conventional primary total knee arthroplasty: clinical and radiological long-term results with a minimum follow-up of ten years Int Orthop 2019 43 06 1345 1354 10.1007/s00264-018-4231-1 30456542

[10] Zhang H Chen J Chen F Que W The effect of tranexamic acid on blood loss and use of blood products in total knee arthroplasty: a meta-analysis Knee Surg Sports Traumatol Arthrosc 2012 20 09 1742 1752 10.1007/s00167-011-1754-z 22065294

[11] Sadigursky D Araujo L M Fernandes R JC Efficacy of Tranexamic Acid in Reducing Blood Loss in Total Knee Arthroplasty Acta Ortop Bras 2018 26 01 63 66 10.1590/1413-785220182601149210 29977148 PMC6025505

[12] Alshryda S Sukeik M Sarda P Blenkinsopp J Haddad F S Mason J M A systematic review and meta-analysis of the topical administration of tranexamic acid in total hip and knee replacement Bone Joint J 2014 96-B 08 1005 1015 10.1302/0301-620X.96B8.33745 25086114

[13] Panteli M Papakostidis C Dahabreh Z Giannoudis P V Topical tranexamic acid in total knee replacement: a systematic review and meta-analysis Knee 2013 20 05 300 309 10.1016/j.knee.2013.05.014 23815893

[14] Yang Z G Chen W P Wu L D Effectiveness and safety of tranexamic acid in reducing blood loss in total knee arthroplasty: a meta-analysis J Bone Joint Surg Am 2012 94 13 1153 1159 10.2106/JBJS.K.00873 22623147

[15] Ahmed I Chawla A Underwood M Infographic: Time to reconsider the routine use of tourniquets in total knee arthroplasty surgery Bone Joint J 2021 103-B 05 828 829 10.1302/0301-620X.103B5.BJJ-2021-0545 33934643

[16] Stimson L N Steelman K R Hamilton D A Chen C Darwiche H F Mehaidli A Evaluation of Blood Loss in Conventional vs MAKOplasty Total Knee Arthroplasty Arthroplast Today 2022 16 224 228 10.1016/j.artd.2022.06.003 35880226 PMC9307488

[17] Fu X She Y Jin G Comparison of robotic-assisted total knee arthroplasty: an updated systematic review and meta-analysis J Robot Surg 2024 18 01 292 10.1007/s11701-024-02045-y 39052153 PMC11272701

[18] Fuji T Fujita S Tachibana S Kawai Y A dose-ranging study evaluating the oral factor Xa inhibitor edoxaban for the prevention of venous thromboembolism in patients undergoing total knee arthroplasty J Thromb Haemost 2010 8 11 2458 2468 10.1111/j.1538-7836.2010.04021.x 20723033

[19] Pereira R T Pentrado J PR Bernardinetti M Nogueira N IA Tavares J P Batista G J Modern orthopedics: the use of robotics in total knee arthroplasty Res Soc Dev 2022 11 12 e521111234960 10.33448/rsd-v11i12.34960

[20] Xu J Li L Fu J Early clinical and radiographic outcomes of robot-assisted versus conventional manual total knee arthroplasty: a randomized controlled study Orthop Surg 2022 14 09 1972 1980 10.1111/os.13323 35848154 PMC9483055

[21] Yi Z Yan L Haibo S Effects of tourniquet use on clinical outcomes and cement penetration in TKA when tranexamic acid administrated: a randomized controlled trial BMC Musculoskelet Disord 2021 22 01 126 10.1186/s12891-021-03968-5 33517881 PMC7847577

[22] Hoeffel D P Daly P J Kelly B J Giveans M R Outcomes of the First 1,000 Total Hip and Total Knee Arthroplasties at a Same-day Surgery Center Using a Rapid-recovery Protocol J Am Acad Orthop Surg Glob Res Rev 2019 3 03 e022 10.5435/JAAOSGlobal-D-19-00022 31157316 PMC6484945

[23] Courtney P M Boniello A J Berger R A Complications Following Outpatient Total Joint Arthroplasty: An Analysis of a National Database J Arthroplasty 2017 32 05 1426 1430 10.1016/j.arth.2016.11.055 28034481

[24] Wang J C Piple A S Hill W J Computer-navigated and robotic-assisted total knee arthroplasty: increasing in popularity without increasing complications J Arthroplasty 2022 37 12 2358 2364 10.1016/j.arth.2022.06.014 35738360

[25] Ofa S A Ross B J Flick T R Patel A H Sherman W F Robotic total knee arthroplasty vs conventional total knee arthroplasty: a nationwide database study Arthroplast Today 2020 6 04 1001 1.008E6 10.1016/j.artd.2020.09.014 33385042 PMC7772451

[26] Itou J Kuwashima U Itoh M Okazaki K Robotic-assisted total knee arthroplasty is not associated with increased risk of postoperative deep vein thrombosis J Exp Orthop 2023Jun 29;10 01 65 10.1186/s40634-023-00628-6 37382867 PMC10310643

[27] Kayani B Konan S Tahmassebi J Pietrzak J RT Haddad F S Roboticarm assisted total knee arthroplasty is associated with improved early functional recovery and reduced time to hospital discharge compared with conventional jig-based total knee arthroplasty: a prospective cohort study Bone Joint J 2018 100-B 07 930 937 10.1302/0301-620X.100B7.BJJ-2017-1449.R1 29954217 PMC6413767

[28] Aggarwal V A Sun J Sambandam S N Outcomes following robotic assisted total knee arthroplasty compared to conventional total knee arthroplasty Arch Orthop Trauma Surg 2024 144 05 2223 2227 10.1007/s00402-024-05231-7 38386067

